# Conjugated detergent micelles as a platform for IgM purification

**DOI:** 10.1002/bit.28089

**Published:** 2022-04-04

**Authors:** Gunasekaran Dhandapani, Ellen Wachtel, Ishita Das, Mordechai Sheves, Guy Patchornik

**Affiliations:** ^1^ Department of Chemical Sciences Ariel University Ariel Israel; ^2^ Faculty of Chemistry Weizmann Institute of Science Rehovot Israel

**Keywords:** conjugated micelles, hydrophobic amino acids, IgM purification, ligand free, [metal:chelator] complexes, nonchromatographic

## Abstract

Immunoglobulin M (IgM) antibodies hold promise as anticancer drugs and as agents for promoting immune homeostasis. This promise has not been realized due to low expression levels in mammalian cells producing IgM class antibodies, and the failure of protein A chromatography for IgM purification. Here, we describe a nonchromatographic platform for quantitatively capturing IgMs at neutral pH, which is then recovered with 86%–94% yield and >95% purity at pH 3. The platform contains micelles conjugated with the [(bathophenanthroline)_3_:Fe^2+^] amphiphilic complex. Inclusion of amino acid monomers, for example, phenylalanine or tyrosine, during conjugation of detergent micelles, allows subsequent extraction of IgMs at close to neutral pH. With the successful implementation of this purification platform for both polyclonal humans and bovine IgMs, we anticipate similar results for monoclonal IgMs, most relevant for the pharmaceutical industry.

## Graphical abstract

Immunoglobulin M (IgM) antibodies are rarely used in medicine due to difficulties associated with their expression and purification. A potentially (a) general, (b) nonchromatographic, (c) ligand‐free, and (d) simple‐to‐implement platform capable of quantitatively capturing IgMs (pH 7) and recovering these in pure form (>95%) at pH 3 (>95% yield) is presented. Unlike other purification methodologies, specially designed micelles conjugated via amphiphilic [metal:chelator] complexes are used.

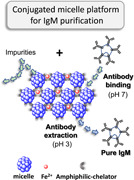

**Abbreviations**
Batho: bathophenanthroline
CD: circular dichroism
DDM: dodecyl β‐d‐maltopyranoside
DDW: double distilled water
DMSO: dimethyl sulfoxide
DLS: dynamic light scattering
IgG: immunoglobulin G
IgM: immunoglobulin M
Ile: isoleucine
Leu: leucine
OG: octyl‐β‐d‐glucopyranoside
PEG: polyethylene glycol
Phe: phenylalanine
SDS‐PAGE: sodium dodecyl sulfate‐polyacrylamide gel electrophoresis
Tyr: tyrosine
Val: valine

## 1 INTRODUCTION

Immunoglobulin‐Ms (IgMs) are the first antibodies produced during immune response in vertebrates (Fellah et al., 1992). They are bound to B‐cell membranes or are secreted, primarily to the blood circulation (Fuentes‐Panana et al., 2004). Whereas membrane‐bound IgM is dimeric, IgM in the blood is pentameric (~900 kDa) (Ehrenstein & Notley, 2010) or hexameric (~1050 kDa), lacking the joining chain (Randall et al., 1992). The three‐dimensional (3D) structure of IgMs was investigated by X‐ray solution scattering and electron microscopy imaging (Pan et al., 2021; Perkins et al., 1991), as well as with cryo‐atomic force microscopy imaging. These indicated that pentameric human IgMs have a mushroom shape with a protruding center that may be responsible for the 10^3^ greater avidity of IgMs toward complement component, C1q (Czajkowsky & Shao, 2009) compared to immunoglobulin G (IgGs). Ten binding domains per pentameric IgM allow parallel binding of more cell‐surface targets with a single antibody. Their large size and structural complexity (Keyt et al., 2020) lead to maximum protein titers in mammalian cell expression, ranging between 0.7 and 0.9 mg/ml (Tchoudakova et al., 2009), a 10–20‐fold reduction compared to IgGs (Keyt et al., 2020).

Purification of IgMs is challenging. Protein A, the gold standard affinity ligand for IgG purification, does not bind IgMs (Keyt et al., 2020). IgMs exhibit lower water‐solubility and a greater tendency to denature under acidic elution conditions compared to IgGs (García‐González et al., 1988; Middaugh & Litman, 1977). These physiochemical properties of IgMs restrict the range of working conditions that can be applied. IgM's high molecular weight (MW) and size (40 × 40 nm^2^; Keyt et al., 2020) translates into a diffusion constant (*K* ^diff^ = 2.6 × 10^−7^ cm^2^/s), that is, approximately half that of IgGs, and limited access to commonly used porous, particle‐based chromatographic media. These characteristics of all IgMs are responsible for overall low recovery yields. Although laboratory‐scale methods for IgM purification do exist (Aoyama & Chiba, 1993; Gagnon et al., 2011; Nethery et al., 1990; Nevens et al., 1992; Steindl et al., 1987; Tchoudakova et al., 2009), none of these purification strategies is currently available on an industrial scale with an associated good manufacturing practice regulatory file (Keyt et al., 2020).

The objective of this communication is to present an alternative purification method that would be straightforward to implement, nondenaturing, and would not be compromised by the large size and small diffusion coefficient of IgMs. Accordingly, we studied a nonchromatographic, ligand‐free method that has demonstrated its utility with IgGs and F(ab′)_2_ fragments: the active medium is based on micellar aggregates that are formed upon conjugation with amphiphilic [(bathophenanthroline)_3_:Fe^2+^] complexes (Dhandapani, Howard, et al., 2019; Dhandapani, Nair, et al., 2019; Dhandapani et al., 2020, 2021) Such aggregates were found to: (i) quantitatively capture IgGs (Dhandapani, Howard, et al., 2019; Dhandapani, Nair, et al., 2019; Dhandapani et al., 2020), as well as the F(ab′)_2_ domain of a monoclonal antibody (Dhandapani et al., 2021) (presumably due to hydrophobic interactions with the detergent aggregates, in agreement with diverse studies showing how IgGs are purified via hydrophobic interaction chromatography) (Follman & Fahrner, 2004; Ghosh & Wang, 2006; Guse et al., 1994; Manzke et al., 1997); (ii) reject hydrophilic impurities; and (iii) allow efficient recovery of pure antibodies from the detergent aggregates at pH 3.8.

**Figure 1:**
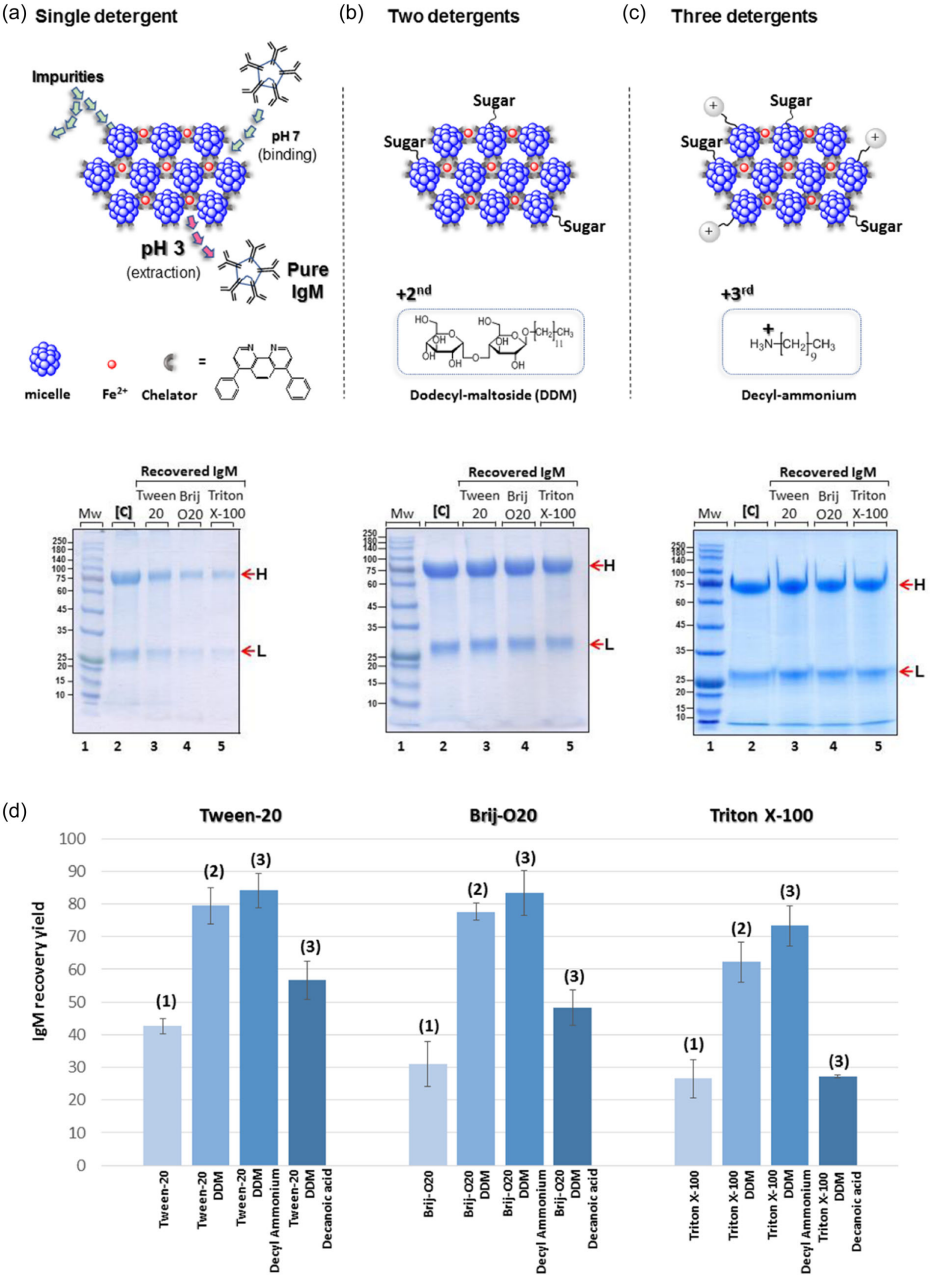
Purification of bovine IgM with conjugated detergent micelles. IgMs captured at pH 7 using micelle aggregates containing one (a), two (b), or three (c) detergents conjugated by the [(bathophenanthroline)_3_:Fe^2+^] complex. Coomassie blue‐stained gels (a–c) show IgM extraction efficiency at pH 3 using indicated detergent combinations. Lane 1, MW markers; lane 2, total IgM added; lanes 3–5, IgM recovered at pH 3. (d) Overall process yield, using Tween‐20, Brij‐O20, or Triton X‐100 plus one or two smaller surfactants. Four replicates were performed. H, L, heavy and light chains; IgM, immunoglobulin M; MW, molecular weight.

Detergent micelle aggregates were prepared as described in the Methods and Materials section. Purifying bovine IgM via the conjugated micelle aggregate protocol included two major steps: In step I, IgMs bind to micellar aggregates at pH 7 and are pelleted with the aggregates (at 21,000*g* for 5 min). Unbound IgMs and impurities present in the supernatant are excluded by pipetting. In step II, bound IgMs are extracted from the aggregates at pH 3 without parallel aggregate dissolution or coextraction of impurities. This two‐step protocol, studied with either Tween‐20, Brij‐O20, or Triton X‐100 as the single detergent, led to 28%–42% recovery yields (Figure 1a, lanes 3–5), while all IgM antibodies were quantitatively captured (Figure S1A, lanes 6–8). However, when a second detergent, either dodecyl β‐d‐maltopyranoside (DDM), containing a maltose headgroup (Figure 1b), or octyl‐β‐d‐glucopyranoside (OG) (Figure S1B), containing a glucose headgroup, was added, this addition significantly improved the recovery yield of bovine IgM to 64%–78% with each of the detergents listed above (Figure 1b).

These findings suggested that mixed detergent micelles presenting sugar headgroups (glucose or maltose) reduce the hydrophobic attraction of IgMs to the micelle aggregates, resulting in improved extraction yield, as observed. We further found that the presence of a third detergent decyl‐ammonium plus either Tween‐20 or Brij‐O20, but not Triton X‐100, further increased extraction yield to 86%–94% (Figure 1c). Systematic supporting evidence for the superiority of three detergents with different head groups, over one or two, is shown in Figure 1d.

Purification trials performed in the presence of a contaminating background were conducted. When bovine IgM was purified from its mixture with *Escherichia coli* lysate, the purity of the recovered IgM was very high (>95%, by densitometry) and the contribution of DDM over other secondary detergents was evident (Figure S2A,B). Additional purification trials in the presence of *E. coli* lysate and with decyl‐ammonium as the third detergent achieved 91%–94% overall yield (Figure S2C). Polyclonal human IgMs were subjected to the optimized “three‐detergent protocol” and analogous behavior was observed (not shown).

**Figure 2:**
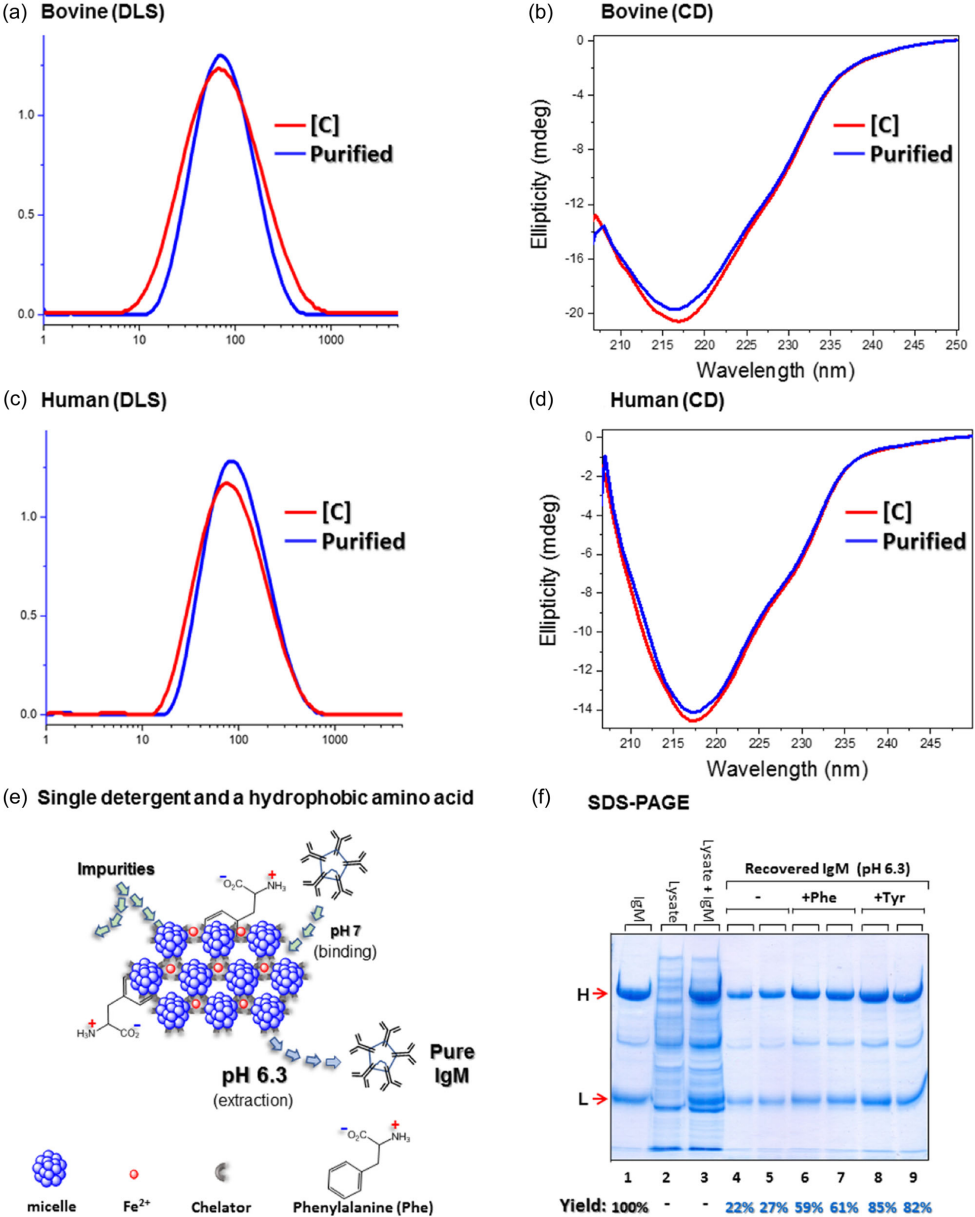
(a–d) DLS and CD analysis of purified bovine and human IgMs. IgMs captured at pH 7 with detergent aggregates containing Tween‐20, DDM, and decyl‐ammonium conjugated with the [(bathophenanthroline)_3_:Fe^2+^] amphiphilic complex, extracted at pH 3. (e) Extraction at pH 6.3. Aggregates containing Tween‐20, the [(bathophenanthroline)_3_:Fe^2+^] amphiphilic complex, and Phe as a platform for IgM capture and extraction. (f) Lanes 1–3: total IgM; total *Escherichia coli* lysate; or both, respectively; lanes: 4–5, 7–8, and 9–10—IgM recovered from Tween‐20 aggregates containing the [(bathophenanthroline)_3_:Fe^2+^] amphiphilic complex with or without Phe or Tyr added during aggregate preparation. Overall process yields shown below the gel were calculated by densitometry using the ImageJ (NIH) program. Gels are Coomassie‐stained. CD, circular dichroism; DDM, dodecyl β‐d‐maltopyranoside; DLS, dynamic light scattering; H, L, heavy and light chains; IgM, immunoglobulin M; Phe, phenylalanine; SDS, sodium dodecyl sulfate‐polyacrylamide gel electrophoresis; Tyr, tyrosine.

Dynamic light scattering (DLS) demonstrated that upon completion of purification, both bovine and human IgMs are individual pentamers: no difference in particle size was observed when compared to the as‐received IgMs that had not encountered any detergent (Figure 2a,c). Circular dichroism (CD) measurements of purified IgMs displayed a negative ellipticity band at ~218 nm characteristic of the antiparallel, β‐sheet secondary structure of IgMs and of other Igs (Steindl et al., 1987) as well (Figure 2b,d). Thus, the purification method presented here was found to preserve the native structure of both human and mouse IgMs.

Since some IgMs undergo denaturation and aggregation at very low pH (Hennicke et al., 2017) extraction of IgMs was also studied at pH 6.3 (Figure 2e). Achieving this goal required the preparation of conjugated detergent micellar aggregates with which captured IgMs would interact more weakly, and hence, a fundamental change in the aggregate chemical composition was essential. We found that the inclusion of either leucine, isoleucine, valine, phenylalanine, or tyrosine during conjugation of Tween‐20 micelles with the [(bathophenanthroline)_3_:Fe^2+^] complexes and the formation of detergent aggregates significantly improved the extraction efficiency of bovine IgMs at pH 6.3 (Figure S3A,B). Best overall yields (~80%) were observed when tyrosine was present (Figure S3A, lanes 7 and 8). Repetition of the latter with *E. coli* lysate as contaminating background (Figure 2f) gave similar results (Figure 2f, lanes 8 and 9). Although the possibility of not exposing IgMs to harsh acidic conditions is a major advantage of our purification platform, extraction at pH 6.3 may suffer from an inability to inactivate viruses, which may be present in the system (Mazzer et al., 2015; Valdés et al., 2002). Therefore, an additional step may be required to assure viral removal from purified IgMs. We note, however, that detergent‐based strategies are used as an alternative to acidic viral inactivation in IgM downstream processing (Keyt et al., 2020). Therefore, the fact that the purification platform described here is composed of detergents, is indeed encouraging.

## 2 MATERIALS AND METHODS

IgM from bovine serum (Sigma; I8135), IgM from human serum (Sigma; I8260), leucine (Sigma; L8000), valine (Sigma; V0500), isoleucine (Sigma; I2752), tyrosine (Sigma; T3754), phenylalanine (Sigma; P2126), iron (II) chloride tetrahydrate (Sigma; F2130), sodium chloride (Sigma; S7653), polysorbate 20 (Tween‐20) (Sigma; 44112), Brij O‐20 (Sigma; 436240), Triton X‐100 (Sigma; laboratory grade), poly (ethylene glycol) 6000 (Sigma; 81260), and Ex‐CELL 610‐HSF medium (Sigma; 14610C). Glycine (Bio‐lab; 07132391), Tris(hydroxymethyl)aminomethane (Bio‐lab; 20092391), MW markers (Bio‐lab; Supermarker2700), bathophenanthroline (GFS Chemicals; C038446), DDM (Carbosynth; DD06199), and octyl OG (Carbosynth; DO05161).

### 2.1 Methods

#### 2.1.1 Preparation of single detergent micelle aggregates

Detergent aggregates were obtained by mixing equal volumes of medium A and B as follows: medium A was prepared by the addition of 3.5 μl of the hydrophobic chelator bathophenanthroline (50 mM in dimethyl sulfoxide (DMSO)‐HCl solution) to 45 μl of a single or a mixture of the following detergents: 0.5 mM Tween‐20; 0.2 mM Brij O20; 0.2 mM Triton X‐100 all in DDW with vigorous vortexing to a final volume of 48.5 μl. An equal volume of medium B, containing 2.5 mM FeSO_4_ in 20 mM NaCl was then added to medium A with vigorous vortexing. After 5 min of incubation at 25°C, 13 μl of 1 M of NaCl was added. After an additional brief incubation (5 min, 25°C), the system was centrifuged for 5 min (relative centrifugal force (21,000*g*, using Microfuge: 5424‐R Eppendorf). The supernatant was discarded and the pellets were briefly washed with 30 μl of cold 20 mM NaCl.

#### 2.1.2 Preparation of two or three detergent micelle aggregates

Preparation of two or three detergent micelle aggregates was accomplished by adding 6.5 μl of 30 mM DDM (in DDW) (for a two‐detergent system) and 3.5 μl of 50 mM decyl‐ammonium (in 100 mM Tris, pH 7.5) (for a three‐detergent system) before addition of the chelator to medium A. Preparation of detergent micelle aggregates composed of a single detergent and a single hydrophobic amino acid was achieved by including 3.8 μl of one of 200 mM phenylalanine, tyrosine, isoleucine, leucine, or valine in DMSO.

### 2.2 Purification protocol

Purification of either human or bovine IgMs was performed on the 100 μl scale. Freshly prepared detergent aggregates were resuspended in 20 μl of serum‐free medium (Ex‐CELL, 610‐HSF), 60 μl of the target IgM (1 mg/ml), and 20 μl of 30% of PEG‐6000. After 10 min of incubation at 25°C, the mixture was centrifuged (21,000*g* for 5 min), the supernatant was discarded, and pellets were briefly washed with 30 μl of cold 20 mM NaCl. An additional identical centrifugation step followed, the supernatant was removed, and the remaining pellet was subjected to extraction conditions. Washed pellets were resuspended with 100 μl of 50 mM Gly (pH 3) in 30 mM NaCl for 15 min at 25°C. An identical centrifugation step was applied and the supernatant was collected for further analysis. When extraction was performed at pH 6.3, 200 mM Tris (at pH 6.3) in 30 mM NaCl was used.

### 2.3 Sodium dodecyl sulfate‐polyacrylamide gel electrophoresis (SDS‐PAGE)

Recovered IgMs were mixed with a sample buffer (4×) containing β‐mercaptoethanol and boiled for 5 min at 95°C. Aliquots (20 µl) were loaded onto a 10% bis‐Tris SDS‐polyacrylamide gel (1 mm thickness) for 90 min at a constant rate of 120 V. All gels were stained with Coomassie Brilliant Blue G‐250. Bands present in Coomassie‐stained gels were quantified using the ImageJ (NIH) standalone version 1.51k.

### 2.4 DLS

Recovered bovine or human IgM (0.3–0.5 mg/ml) and as‐received, pure IgM, as a control, were dissolved with 50 mM glycine (pH 3) in 30 mM NaCl. Samples were centrifuged (21,000*g*, 20 min) and the supernatant was used for analysis. The intensity‐weighted size distributions of bovine and human IgM samples were determined using the autocorrelation spectroscopy protocol of the Nanophox instrument (Sympatec GmbH).

### 2.5 CD spectroscopy

Recovered bovine and human IgM were subjected to CD analysis using a Chirascan CD spectrometer (Applied Photophysics). CD spectra report ellipticity (*θ*), proportional to the difference in absorbance of left and right circularly polarized light [*θ* = 3300° (AL − AR)] as a function of wavelength. A quartz cell of path length 0.1 cm was used for the measurement. The CD spectra were recorded with 2 nm bandwidth resolution in 1 nm steps at 25°C. The collected CD spectra were corrected for baseline distortion by subtracting a reference spectrum of the corresponding buffer solution.

### 2.6 Densitometry

Bands present in Coomassie‐stained gels were quantified using the EZQuant program (http://www.ezquant.com/en/). Process yield was calculated by comparing the intensity of bands representing a known amount of calibrated, purified target to the recovered target at the end of the purification process.

## 3 CONCLUSION

IgMs are captured quantitatively at neutral pH and recovered at 86%–94% yield and >95% purity at pH 3 using three detergent micelles conjugated with the [(bathophenanthroline)_3_:Fe^2+^] amphiphilic complex. IgM extraction at pH 6.3 succeeds if tyrosine or phenylalanine is added during Tween‐20 micelle aggregate formation. Optimization of this nonchromatographic, ligand‐free purification platform will now be directed toward monoclonal IgMs, which are relevant for the pharmaceutical industry.

## Supporting information

Supporting information.
