# Supplementary material for: Conjugated detergent micelles as a platform for IgM purification
Source: Biotechnol Bioeng. 2022 Apr 4;119(7):1997–2003. doi: 10.1002/bit.28089 (PMC9325453; doi:10.1002/bit.28089)
Supplement: Supplementary file 1 — Supporting information. [file BIT-119-1997-s001.docx]

**Conjugated Detergent Micelles as a Platform for IgM Purification**

Gunasekaran Dhandapani,^1^ Ellen Wachtel,^2^ Ishita Das,^2^ Mordechai Sheves ^2^ and Guy Patchornik^1*^

**^Supplementary^**

^1^ Department of Chemical Sciences, Ariel University, 70400, Ariel, Israel.

^2^ Faculty of Chemistry, Weizmann Institute of Science, 76100, Rehovot, Israel.

*Corresponding author:

Email: [guyp@ariel.ac.il](mailto:guyp@ariel.ac.il)

**
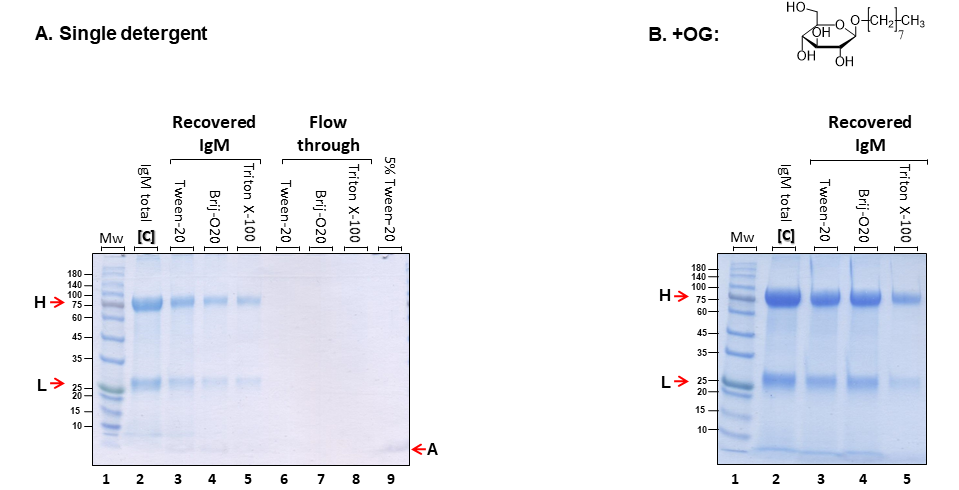
**

**Figure S1 caption: Purification of bovine IgM with micelle aggregates containing one or two detergents. A.** Lane 1 - Molecular weight markers (MW); lane 2: Total IgM present; lanes 3-5: IgM recovered from micelle aggregates conjugated with the bathophenanthroline (batho):Fe^2+^ amphiphilic complex; lanes 6-8 supernatant composition following IgM capture, using the single detergent micelle aggregates; lane 9 - band intensity represents 5% of Coomassie stained Tween-20 aggregates. **B.** **Impact of a second detergent: octyl glucoside (OG)** **on IgM extraction.** Lane 1 - Molecular weight markers (MW); lane 2 - Total IgM present; lanes 3-5, IgM recovered from Tween-20 aggregates containing a second detergent: OG. **H**, **L** identify **H**eavy and **L**ight chains of the IgM while **A** identifies the detergent **A**ggregates. Gels are Coomassie stained.

**
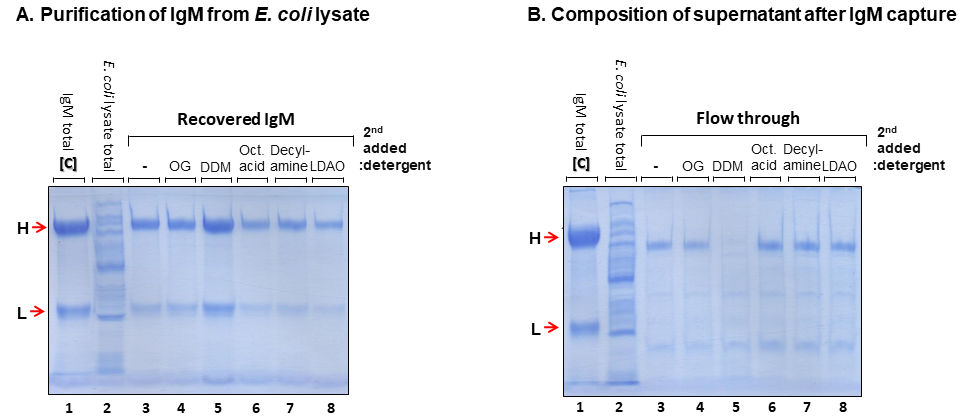
**

**
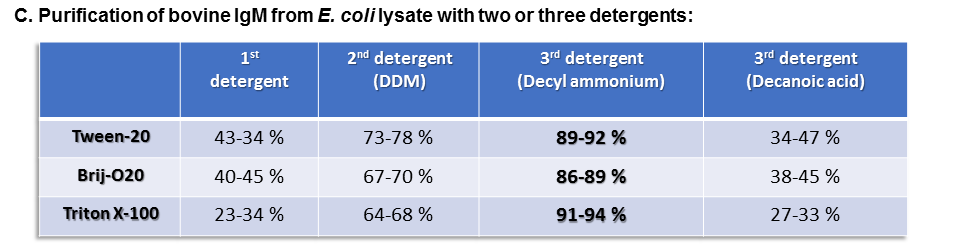
**

**Figure S2 caption: Purification of bovine IgM from *E. coli* lysate using aggregates comprising one, two or three detergents and extraction at pH 3. A.** Lane 1 - Total IgM present; lane 2 - Total *E. coli* lysate present; lanes 3-8: IgM recovered with detergent aggregates comprised of: [Tween-20:batho:Fe^2+^] with the addition of a second detergent: OG, DDM, octanoic acid, decylamine and LDAO, respectively. **B.** As in **A**, showing the composition of the supernatant after IgM capture (*i.e.*, the flow through). **H**, **L**, represent the **H**eavy and **L**ight chains of IgM, respectively. Gels are Coomassie stained. **C.** Summary of process yields using different detergent combinations by densitometry.


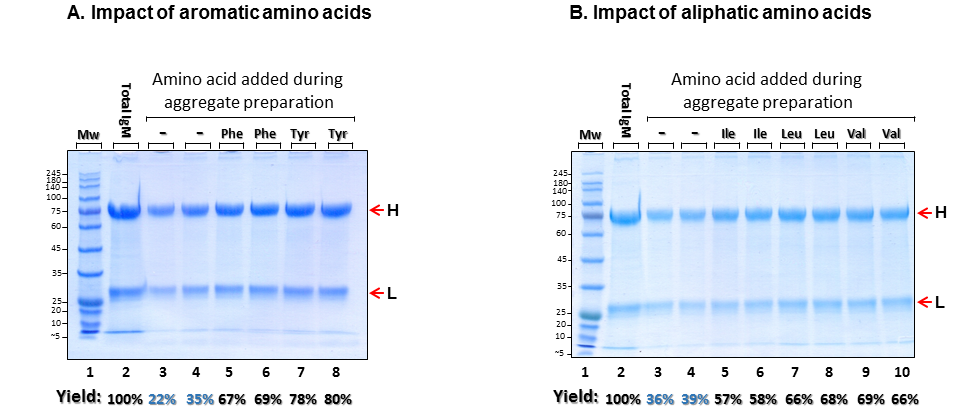


**Figure S3 caption: Effect of hydrophobic amino acid addition. A-B.** Tween-20 aggregates were prepared with or without the addition of aromatic or aliphatic amino acids. These additions were necessary to capture bovine IgM at pH 7 and to extract the antibody at pH 6.3. Lane 1 - molecular weight (MW) markers; lane 2: total IgM present; lanes 3-4, 5-6 and 7-8 quantitate recovered IgM. Overall process yields shown below the gels were calculated by densitometry using the ImageJ (NIH) program. Gels are Coomassie stained.
